# Effect of sildenafil (Revatio) on postcardiac surgery acute kidney injury: a randomised, placebo-controlled clinical trial: the REVAKI-2 trial protocol

**DOI:** 10.1136/openhrt-2018-000838

**Published:** 2018-10-18

**Authors:** Hardeep Aujla, Tracy Kumar, Marcin Woźniak, William Dott, Nikol Sullo, Lathishia Joel-David, Thomas Morris, Cassandra Brookes, Shaun Barber, Gavin James Murphy

**Affiliations:** 1 Department of Cardiovascular Sciences and NIHR Leicester Biomedical Research Centre, Cardiovascular Theme, University of Leicester, Glenfield Hospital, Leicester, UK; 2 Leicester Clinical Trials Unit, University of Leicester, Leicester General Hospital, Leicester, UK

**Keywords:** cardiac surgery, renal disease, pharmacology, endothelium

## Abstract

**Introduction:**

Acute kidney injury (AKI) is a common and severe complication of cardiac surgery. The administration of pharmacological renoprotective agents during the perioperative period could prevent or reduce the severity of AKI and improve clinical outcomes. Experimental studies suggest that sildenafil may have therapeutic potential for the prevention of AKI. This trial will test the hypothesis that postoperative AKI will be reduced in cardiac surgery patients if they receive sildenafil compared with placebo.

**Methods and analysis:**

Adult cardiac surgery patients 18 years of age or above undergoing cardiac surgery with cardiopulmonary bypass and cardioplegic arrest at a single tertiary cardiac centre in the UK will be randomised in a 1:1 ratio to receive either sildenafil or placebo. The primary outcome is serum creatinine concentration measured at preoperation and daily for up to 7 days postoperatively. Secondary outcomes will include measures of inflammation, organ injury, volumes of blood transfused and resource use. Allocation concealment, internet-based randomisation stratified by operation type, and blinding of outcome assessors will reduce the risk of bias. A sample size of 112 patients will have a 90% power to detect a mean difference of 10 μmol/L for serum creatinine values between treatment and placebo control groups with an alpha value of 0.05.

**Ethics and dissemination:**

The trial protocol was approved by a UK ethics committee (reference 15/YH/0489). The trial findings will be disseminated in scientific journals and meetings.

**Trial registration number:**

ISRCTN18386427.

**Key questions**

**What is already known about this subject?**

- An acute decline in kidney function resulting in increased postoperative complications is common among patients following cardiac surgery. To date, there is no evidence to suggest that currently available pharmacological interventions offer effective prevention.
- Studies in animal models suggest that sildenafil can have renoprotective effects during cardiac surgery. Similar doses in humans can be administered without adverse side effects.

**What does this study add?**

- The objective of the REVAKI-2 trial is to determine whether postoperative AKI will be reduced in cardiac surgery patients following administration of sildenafil.

**How might this impact on clinical practice?**

- Improving outcomes in patients with AKI is a clinical research priority as the population ages and patients with more comorbidities are referred for cardiac surgery. If effective, this intervention could be widely adopted.

## Introduction

### The clinical problem

Acute kidney injury (AKI) manifests clinically as an acute reduction in glomerular filtration rate (GFR) and is a common and severe complication of cardiac surgery.^1^ It affects up to 30% of patients in contemporary studies and is associated with a fourfold increase in perioperative mortality.^1–3^ As the population is ageing and elderly patients with more comorbidities are increasingly referred for cardiac surgery,^4^ it is predicted that the risk of AKI will increase. Despite the importance of this clinical problem, our understanding of the underlying processes is poor and there is no effective treatment.^5^ In 2012, improving outcomes in patients with AKI was recognised as a clinical research priority by England’s National Health Service.^6^

### Renal injury and adverse outcome in cardiac surgery

AKI in cardiac surgery commonly occurs within a well-defined perioperative period that includes surgery and the use of cardiopulmonary bypass (CPB). Many investigators have tested the hypothesis that concomitant administration of a pharmacological renoprotective agent during the perioperative period should prevent or reduce the severity of AKI and improve clinical outcomes. However, to date, none of these strategies have demonstrated significantly improved clinical outcomes. A recent Cochrane meta-analysis that evaluated all pharmacological strategies for the prevention of AKI concluded that there was no evidence to suggest that currently available pharmacological interventions during surgery can protect the kidneys from damage.^5^ The adverse consequences of developing AKI and the lack of any effective therapy justify the evaluation of new pharmacological interventions for the prevention of AKI in clinical studies.

### Pathophysiological process of AKI

One explanation for the failure of current strategies to improve outcomes in patients with AKI is that our understanding of the pathophysiological processes underlying AKI is poor. Much of our current knowledge is based on studies of warm ischaemic injury in rodents. There are significant differences in renal physiology and in the renal response to injury between rodents and humans, and consequently, renoprotective strategies developed in rodent models have failed to translate into clinical benefits.^7 8^ This has led to calls for the development of large animal models of renal dysfunction with closer homology to humans to facilitate more effective translation.^7 8^ To address this, we developed a novel large animal (porcine) model of post-CPB AKI that shows significant homology to that described in humans.^9^ AKI in this model is associated with renal endothelial dysfunction, medullary hypoxia and depletion of high-energy phosphates as well as reduced nitric oxide (NO) bioavailability.^10 11^ We observed that interventions that attenuated post-CPB AKI in this model, red cell transfusion or administration of an endothelin A receptor antagonist, were associated with increased NO bioavailability. We then tested the hypothesis that augmentation of endogenous NO pathways may represent a novel therapeutic strategy for the prevention of AKI in the porcine model. Endogenous NO bioavailability is augmented by phosphodiesterase type-5 (PDE-5 inhibitors), which prevent the breakdown of cyclic guanosine monophosphate (cGMP), a secondary messenger of NO pathways.^12^ In experimental studies, PDE-5 inhibition attenuates ischaemic AKI in rats^13^ and reduces vasoconstriction in autotransplanted porcine kidneys.^14^ We demonstrated that in the porcine post-CPB AKI model, sildenafil increased NO bioavailability and prevented endothelial dysfunction, glomerular inflammation and reduced renal clearance.^15^ This occurred at a dose (10 mg as a single bolus at the commencement of CPB) that did not produce any effect on pulmonary compliance or pulmonary vascular endothelial function. The similarities between porcine and human renal anatomy, haemodynamics and function,^16–18^ and the quantitative and qualitative homology between post-CPB AKI in humans and pigs^9 10^ supported the translation of findings in this model into clinical trials.

### Sildenafil as a renoprotective intervention in cardiac surgery

Intravenous sildenafil (Revatio; Pfizer) is a phosphodiesterase (PDE) type 5 inhibitor that acts by inhibiting the breakdown of cGMP, a NO secondary messenger. By upregulating NO activity, it reverses endothelial dysfunction in settings such as diabetes and other inflammatory diseases and has proven clinical efficacy in the management of erectile dysfunction (as Viagra), diabetic cardiomyopathy^19^ and pulmonary hypertension.^20^ Intravenous sildenafil is currently licensed for the treatment of pulmonary hypertension and has orphan designation (EU/3/10/815) for the treatment of postcardiotomy right ventricular failure following cardiac surgery.^21^ In a preliminary dose funding study (REVAKI-1), we demonstrated that sildenafil at a dose of 12.5 mg can be administered safely to patients undergoing cardiac surgery.^22^ This dose achieved plasma concentrations that are clinically effective in humans taking the oral formulation,^23 24^ and similar to the levels observed in the porcine preclinical study, without adverse side effects. Sildenafil administered as a bolus of 10 mg over 10 min followed by an infusion of 2.5 mg over 2 hours did produce a transient drop in blood pressure in some patients; however, this was transient, lasting less than 5 min and was reversible by short-acting vasoconstrictors (phenylephrine, ephedrine) that are routinely used to maintain mean arterial blood pressure within the desired range during anaesthesia. Using this information, we designed a randomised clinical trial to compare this dose with a placebo. The aims of the trial are to compare levels of kidney function and injury in the two groups, as well as markers of injury in other organ systems that may be protected by sildenafil treatment.

### Aims and objectives

The REVAKI-2 Trial tests the hypothesis that postoperative AKI will be reduced in cardiac surgery patients identified as being at increased risk of developing AKI preoperatively, following administration of sildenafil, a PDE-5 inhibitor.

Our secondary hypotheses are that the frequency of postoperative AKI, as defined by Kidney Disease Improving Global Outcomes (KDIGO) criteria, as well as the severity of acute lung and myocardial injury will be reduced in high-risk patients identified as being at increased risk of developing AKI preoperatively, by measuring the effects of sildenafil on platelet, leucocyte and endothelial activation.

Specific objectives of this trial are to:

1. Estimate the difference in serum creatinine levels (measured daily for up to 7 days postoperatively and adjusted for baseline values) between patients allocated to the sildenafil and placebo groups.
2. Estimate the difference in the frequency of AKI (defined as a rise in serum creatinine of >26 µmol/L within 48 hours or a doubling of the serum creatinine within 7 days as defined by the KDIGO criteria)^4^ between patients allocated to the sildenafil and placebo groups. Individuals who receive renal replacement therapy (RRT), including haemofiltration, are considered to have met the criteria for stage 3 AKI irrespective of the stage they are in at the time of RRT.
3. Estimate the difference in biomarkers of postoperative renal injury, and myocardial injury, between patients allocated to the sildenafil and placebo groups.
4. Estimate the difference in the frequency of sepsis, low cardiac output, acute lung, brain or gut injury or death between patients allocated to the sildenafil and placebo groups. These outcomes, along with the primary outcome will also be considered as a composite endpoint.
5. Estimate the difference in Multiple Organ Dysfunction Scores between patients allocated to the sildenafil and placebo groups.
6. Estimate the difference in the frequency of other adverse events not listed above between patients allocated to the sildenafil and placebo groups.
7. Estimate the median difference in hospital length of stay between patients allocated to the sildenafil and placebo groups.
8. Estimate whether sildenafil increases the frequency of adverse drug reactions; specifically hypotension that requires treatment or allergic responses.
9. Estimate whether the observed effects of sildenafil may be attributable to changes in endothelial function.
10. Estimate whether the observed effects of sildenafil may be attributable to changes in platelet, leucocyte or endothelial cell activation.

## Methods and analysis

### Study design

This is a single-centre, double-blinded, parallel-group, randomised controlled trial of intravenous sildenafil versus placebo.

### Study population and recruitment

The study will be carried out at a single tertiary cardiac surgery centre in the UK. If sildenafil is effective, its clinical benefits and impact on resource use will be most apparent in patients at greatest risk of developing AKI. In the UK, over one-third of patients are at risk of developing AKI.^20 25^ Patients at increased risk of developing AKI will be identified preoperatively using a risk score developed and validated by these investigators in a multicentre population of over 40 000 cardiac surgery patients^1^ that has been designed to enable cohort enrichment for clinical trials on renoprotective interventions in cardiac surgery (http://www.cardiacsurgeryleicester.com/our-research/acute-kidney-injury-risk-score-calculator/).

In the MARACAS study,^26^ using a cut-off of 22% for the score (positive predicted value (PPV 0.55), 1 in 8 patients were eligible, 1 in 15 were recruited and 39/61 patients recruited thus far have developed AKI. The REVAKI-2 trial will use similar eligibility criteria.

#### Inclusion criteria

Patients may enter the study if ALL of the following apply:

1. Adult cardiac surgery patients 18 years of age or above undergoing cardiac surgery with cardiopulmonary bypass and cardioplegic arrest.
2. Identified as representing a high-risk group for AKI using a modified AKI risk score; a predicted risk score of 20% equates to a PPV for developing AKI of >55%.
3. Female subjects of childbearing potential are not to be pregnant (to be confirmed by urine human chorionic gonadotropin pregnancy test prior to dosing). Women are considered not to be of childbearing potential if they have been surgically sterilised (eg, tubal ligation, oophorectomy or hysterectomy) or are postmenopausal (defined as serum follicle-stimulating hormone level of ≥30 IU/mL) in the absence of hormone replacement therapy and complete absence of menses for at least 24 consecutive months.
4. Able, in the opinion of the investigator, and willing to give informed consent.

#### Exclusion criteria

Patients may not enter study if ANY of the following apply:

1. Cardiac surgery patients (<18 years) undergoing cardiac surgery with cardiopulmonary bypass and cardioplegic arrest.
2. Emergency or salvage procedure
3. Ejection fraction <20%.
4. Chronic kidney disease stage 5, defined as estimated GFR (eGFR) <15 mL/min or RRT (as per the Modified diet in Renal Disease formula).
5. Patients with a pre-existing sepsis or organ injury defined as documented sepsis, AKI, acute lung injury, myocardial infarction, low cardiac output, liver injury, stroke or pancreatitis within 5 days of surgery.
6. Administration of potent CYP 3A4 inhibitors within 1 month prior to study participation (eg, HIV protease inhibitors, imidazole antifungals and erythromycin; please see online supplementary appendix 1 for a full list of prohibited medications).
7. Administration of nitrate medicines or NO donors (eg, glyceryl trinitrate or Nicorandil) within 24 hours of surgery.
8. Patients allergic to sildenafil or any other PDE-5 inhibitor.
9. Any ongoing malignancy or prior malignancy that currently requires treatment.
10. Patients who are participating in another interventional clinical study.
11. Patients who have loss of vision in one eye due to non-arteritic anterior ischaemic optic neuropathy, regardless of whether it is connected to previous PDE-5 inhibitor exposure.
12. Risk of pregnancy.
13. Severe hepatic impairment.
14. Severe hypotension (blood pressure <90/50 mm Hg) on the day prior to surgery.
15. Administration of the guanylate cyclase stimulators, such as riociguat.
16. Unable, in the opinion of the investigator, or unwilling to give informed consent.

10.1136/openhrt-2018-000838.supp1 Supplementary data

### Intervention being investigated

#### Treatment regimes

Patients will be screened by the investigators to assess eligibility for entry into the trial. Eligible patients undergoing cardiac surgery with CPB who consent to participate will be randomised in a 1:1 manner to either:

1. Sildenafil.
2. Placebo.

#### Sildenafil

The active trial drug, Revatio, is the citrate salt of sildenafil, a selective inhibitor of cGMP-specific phosphodiesterase type 5 (PDE-5). Sildenafil citrate is designated chemically as 1-[[3-(6,7-dihydro-1-methyl-7-oxo-3-propyl-1*H*-pyrazolo [4,3-*d*] pyrimidin-5-yl)−4-ethoxyphenyl] sulfonyl]-4-methylpiperazine citrate. The active trial medication is supplied in single-use glass vials and as a clear, colourless, sterile, ready-to-use solution containing 10 mg (12.5 mL) of sildenafil in 5% glucose solution. Each millilitre of solution contains 1.124 mg sildenafil citrate, 50.5 mg dextrose and water for injection. Sildenafil is manufactured under Good Manufacturing Practice (EU-GMP) by Pfizer. The protocol for administration of sildenafil and placebo (5% glucose solution) is described in table 1. Deviations from the protocol are defined as the non-administration of the allocated drug or its administration not in accordance with the trial protocol.

**Table 1:** Drug and placebo preparations

	Bolus (syringe 1)		Continuous infusion (syringe 2)	
	Intravenous bolus (dose)	Intravenous bolus (rate)	Intravenous continuous (dose)	Intravenous continuous (rate)
Treatment	12.5 mL (ie, 10 mg of sildenafil). Diluted with 5% glucose to a total of 15 mL Marked as syringe 1	45 mL/h; that is, to run over 20 min	2.5 mg sildenafil as a 2-hour continuous infusion, diluted to a total of 50 mL in 5% glucose Marked as syringe 2	25 mL/hour, that is, to run over 2 hours
Placebo	15 mL 5% glucose Marked as syringe 1	45 mL/hour; that is, to run over 20 min	50 mL 5% glucose Marked as syringe 2	25 mL/hour, that is, to run over 2 hours

### Primary and secondary endpoints

#### Primary outcome

The primary outcome for the trial is serum creatinine measured daily at preoperation, on return to cardiac intensive care unit, 6–12, 24, 48, 72, 96, 120 hours, or if still an inpatient at 144 and 168 hours. The final serum creatinine sample will be measured at 6 weeks.

#### Secondary outcome

The secondary outcome measures are listed in online supplementary table 1.

### End of the trial

The definition of the end of the trial is the date when all participants have completed the 3-month postal follow-up or have been lost to follow-up.

### Clinical management of study participants

#### Concomitant treatment

Patients may receive medications and/or other therapies to treat adverse events as deemed necessary by the investigator or the patient’s physician. Concomitant medications and/or therapy that become necessary during the trial and any changes in concomitant medication and/or therapy will be recorded in the case report forms. Details of concomitant medications and therapy will include generic drug name, dose, route, duration and indication.

#### Preoperative care

Eligible patients will receive standard care preoperatively as per local practice.

#### Anaesthesia and perioperative care

Local protocols for anaesthesia and perioperative care will be used. Anaesthetic maintenance may use either volatile or intravenous anaesthetic agents or both in combination.

Cardiopulmonary bypass will be managed according to local practice. It is expected that patients will undergo non-pulsatile CPB (28°C–35°C), using either a standard or closed venous reservoir, a roller pump, a hollow fibre oxygenator and a non–heparin-bonded circuit. Target flows will be 2.4–2.7 L/min/m^2^, with mean arterial blood pressure (MABP) maintained between 60 and 80 mm Hg. Circuit prime will not be standardised; however, volumes and types of colloid and crystalloid solutions administered will be recorded. Intermittent or continuous, antegrade and/or retrograde blood cardioplegic arrest will be performed. Haematocrit will be maintained >23. Target activated clotting time of >400 s will be achieved with heparin (300 U/kg as a loading dose) for bypass. Heparin reversal will be achieved with the administration of protamine sulfate in a 1:1 ratio as per standard practice.

#### Haemodynamic support

The use of inotropes or vasopressors will be at the discretion of the attending physician. Blood products will be transfused using existing unit protocols. Fluid replacement therapy will be as directed according to local practice.

#### Postoperative care

Intravenous glycopyrolate, atropine, atrial or dual chamber epicardial pacing will be used to achieve a target heart rate (70–110 bpm). The use of inotropes or vasopressors will be at the discretion of the attending physician. Postoperative oliguria, defined as a urine output <0.5 mL/kg/h for four consecutive hours, will be treated initially with fluid boluses to maintain the central filling pressure >12 mm Hg, and then inotropes (enoximone, dobutamine, epinephrine) or pressor agents (norepinephrine or vasopressin) as indicated to maintain adequate perfusion pressure (ie, MABP >80 mm Hg or within 10% of preoperative MABP), or cardiac output (ie, cardiac index >2.1 L/min/m^2^) as determined by appropriate invasive monitoring. Persistent oliguria resistant to these measures may be managed by forced diuresis, using, for example, furosemide. Decisions about discharge from intensive care unit (ICU), high dependency unit (HDU) and from hospital will be made on the basis of existing institutional protocols.

### Research procedures

#### Screening and eligibility assessment

The patients’ AKI risk score will be calculated at the preoperative assessment clinic or from our standard in patient referral protocols that include detailed clinical and demographic information (table 2). A patient information sheet (PIS), approved by the local Research Ethics Committee, will be sent by post to all potentially eligible patients waiting at home. For patients waiting in hospital, the PIS will be given to them in person. The patient will have time to read the patient information sheet and to discuss their participation with others outside the research team (eg, relatives or friends) if they wish. Each patient will have at least 24 hours to consider whether to participate or not. In a few cases, this time interval may be less, for example for patients admitted for urgent surgery without prior notification to the waiting list co-ordinator. It is important to include these patients for the applicability of the trial findings since about 50% of patients having cardiac surgery are admitted as urgent cases, and these are often those at greatest risk for AKI. Written informed consent will be obtained prior to surgery. Details of all patients approached for the trial and reason(s) for non-participation (eg, reason for being ineligible or patient refusal) will be documented by the research staff in the form of a screening log that will not contain patient-identifiable information.

**Table 2:** Key data collection points and procedures

	Preoperation	Operation day	Day 1	Day 2	Day 3	Day 4	Day 5	Discharge	6 weeks	3 months
Eligibility	✓									
Written consent	✓									
Concomitant medication concomitant medication only in relation to infection apart from pre op	✓	✓	✓	✓	✓	✓	✓	✓	✓	✓
Pregnancy testing	✓									
Randomisation	✓									
Operative details		✓*								
MODSs	✓	(6–12 hours)	✓ (24 hours)	✓ (48 hours)	✓ (72 hours)	✓ (96 hours)				
Clinical outcomes			✓	✓	✓	✓	✓		✓†	✓‡
Serious adverse event monitoring/adverse events		✓	✓	✓	✓	✓	✓	✓	✓†	✓
Bloods: serum biochemistry (creatinine, amylase, liver function tests) and full blood counts	✓	✓ (CICU* and 6–12 hours)	✓*	✓*	✓*	✓*	✓*§	✓§*	✓	
Organ injury mark*e*rs; urine NGAL, serum troponin	Urine ✓ Troponin I	✓ Troponin I (6–12 hours)	Urine ✓	✓ Troponin I (48 hours)						
RH-PAT testing	✓		✓							
Bloods: citrated whole blood for flow cytometry	✓	✓ (6–12 hours)		✓						
Tracheal aspirate		✓ (4–6 hours)								
Questionnaire									✓	✓

*Indicates samples taken usually as part of normal care.

†4–6 week time point in accordance with normal postoperative care.

‡Indicates data collection via postal questionnaires.

§Final time point if patient is discharged.

CICU, cardiac intensive care unit; MODS, Multiple Organ Dysfunction Score; NGAL, Neutrophil Gelatinase Associated Lipocalin; RH-PAT, reactive hyperaemia peripheral arterial tonometry.

#### Randomisation and code breaking

**Figure 1:**
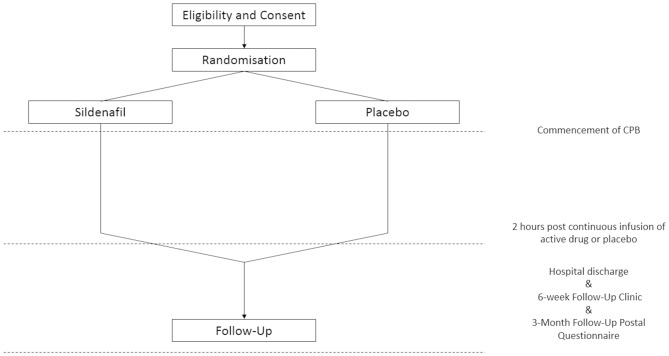
Patient flows showing randomisation, intervention period and follow-up period. CPB, cardiopulmonary bypass.

Patients will be randomly assigned in a 1:1 ratio to either sildenafil or placebo using an internet-based randomisation system (Sealed Envelope, MHRA recognised facility), which will be managed by Leicester Clinical Trials Unit (LCTU). Randomisation will be stratified by (1) type of procedure: coronary artery bypass grafting (CABG), valve, CABG and valve, other; and (b) baseline eGFR: <60, ≥60. Random allocations will be generated only after the relevant baseline data to identify the patient has been entered into the system, guaranteeing concealment of allocation and a definitive log of participants. Patients who consent will be randomised by the unblinded member of the research team. If patients are unexpectedly rescheduled, they will retain their trial numbers and randomised allocation. Detailed instructions for the randomisation process will be provided in a separate manual (figure 1).

#### Trial-specific tests and procedures

Participants will undergo research tests and procedures. These are in addition to tests and procedures undertaken as part of standard of care (SOC). However, if the routine samples are not taken as part of SOC, then these blood samples will be taken for the trial as additional volumes.

A full blood count will be carried out by the hospital laboratory. Serum creatinine and liver function tests will be analysed in the hospital laboratory. Microvesicles, platelets, leucocytes and endothelial cell activation will be analysed by microfluidics and flow cytometry. In addition to these tests, the results of routinely collected arterial blood lactate values will be used to assess the time to clearance of the oxygen debt. These tests are measured in an ad hoc manner as dictated by a range of clinical requirements but as a rule of thumb are measured hourly for the first 4 hours post admission to ICU then every 2–4 hours thereafter until discharge. The frequency of these tests will not be influenced by recruitment in the trial.

Collection of tracheal aspirate, aspirate of tracheal secretions, may be used to measure pulmonary leucocyte extravasation and protein concentrations. At 4–6 hours postsurgery, bronchial suction will be performed as SOC for ventilated patients. If necessary, we will administer 5 mL of normalised saline via the endotracheal tube and aspirate bronchial secretions using an aseptic technique. This aspirate will be analysed for protein concentrations and leucocyte content (flow cytometry). The Endo-Pat 2000 device (Itamar Medical, Caesarea, Israel) will be used to measure reactive hyperaemia peripheral arterial tonometry as an index of regional endothelial function as previously described^27^ at baseline and at 24 hours post-surgery.

#### Planned recruitment rate

We estimate that we will require 2 years to recruit the target population, assuming the cardiac unit in Glenfield will continue to undertake 1200 adult cardiac cases per year. The recruitment rate will equate roughly to one patient per week. We anticipate an additional 6 months for the completion of follow-up and resolution of database queries. This is based on a similar recruitment strategy to a previous observational study.^26^

#### Measures taken to avoid bias

All necessary steps will be taken to reduce the risk of bias.^28 29^ The trial will be analysed on an intention-to-treat basis, that is, outcomes will be analysed according to the treatment allocation, irrespective of future management and events, and every effort will be made to include all randomised patients. Selection bias will be minimised by concealed randomised allocation. Patients, clinicians and researchers will be blinded to the intervention to minimise procedural bias. Detection bias will be minimised by objective source data verification of the data for the primary endpoint and blinding of laboratory staff analysing biomarkers and inflammatory processes. Detection bias for clinical outcomes that are not part of the primary endpoint will be minimised using objective outcome criteria; as defined in the outcome section above and recorded in a blinded manner. Decisions about discharge from ICU, HDU and from hospital will be made by clinical staff based on existing institutional protocols. ICU/HDU transition will be defined as transition from level 3 (1:1 nursing ratio) to level 2 (1:2 nursing ratio). HDU/ward transition will be defined as time of arrival on the ward. To minimise attrition bias, we aim to include data for all randomised participants in the data analyses.

#### Adverse events

Adverse events will be recorded and reported in accordance with the University of Leicester’s and University Hospitals Leicester NHS Trust’s policies for reporting research-related adverse events. In cardiac surgery, postoperative transient complications are not unexpected and are not infrequent. The research team will only notify deaths and ‘unexpected’ non-fatal serious adverse events (SAEs) to the Trial Sponsor (University of Leicester Research Office). The sponsor will inform the research team which SAEs should be reported to the research ethics committee.

### Statistical analyses

The primary outcome for the trial will be the serum creatinine values measured postoperatively, adjusted for baseline values. On the basis that observed SD for serum creatinine values from the MARACAS trial was 37 μmol/L, and the mean observed correlation between baseline and six postsurgery measures was 0.84, we estimate that a sample size of 56 patients per group will have a 90% power to detect a mean difference of 10 μmol/L for serum creatinine values over six postintervention time points, between treatment and control groups with an alpha value of 0.05. This equates to a moderate effect size. Additionally, the study has 80% power to detect a 33% reduction in the frequency of AKI, as a secondary endpoint. We propose to recruit 126 patients (63 per group) anticipating that 10% of patients will be treated outside of the protocol, withdrawn or lost to follow-up.

AKI is a key secondary endpoint of interest. If the observed KDIGO AKI event rate was 65%, as observed in the MARACAS trial, a sample size of 59 patients per group will allow us to detect an absolute reduction in the frequency of AKI to 40% with an 80% power and 5% significance (two-tailed).

Specific morbidities and other adverse events are too infrequent for the trial to be able to detect differences between the treatment and control groups. Frequencies of these adverse outcomes will be tabulated, in line with guidelines for reporting adverse events in trials.^29^ Our mechanism substudy will be performed on a subgroup of 96 patients. As this analysis is exploratory, no power calculation was performed.

#### Plan of analysis

The primary analysis will take place when follow-up is complete for all patients and will be performed on an intention-to-treat basis. Continuous longitudinal outcomes will be compared using linear mixed-effects methodology with the treatment group and study design variables fitted as fixed effects, and patient terms as random effects. Binary outcomes will be compared between treatment groups using logistic regression. Continuous outcomes will be compared using linear regression. Time to event outcomes will be compared using Cox’s proportional hazards models. All statistics will be reported with 95% CIs.

#### Sensitivity analysis

Two sensitivity analyses are planned, comparing effect estimates for the primary outcome for (1) a per-protocol comparison including only those patients who receive the allocated intervention, that is, excluding patients who receive the incorrect intervention, are withdrawn prior to the intervention, or where there is incomplete compliance with intervention, (2) including only patients with complete follow-up data.

### Trial management

The trial will be managed by the Cardiac Surgery Clinical Trials Team at the University of Leicester, supported by the LCTU, a UK Clinical Research Collaboration registered CTU. The CTU will manage an internet-based randomisation system. They will develop and maintain the trial database, check data quality as the trial progresses and carry out statistical analyses in collaboration with the clinical investigators.

### Patient and public involvement

The Leicester Cardiac Surgery (LCS) Patient and Public Involvement (PPI) group brings together cardiac patients, some of whom have participated in clinical trials, and members of the public, many of whom have PPI clinical research experience in local and national organisations. LCS PPI group members actively participate in research activities. A consultation exercise with the entire PPI group has informed the study design and selection of clinical endpoints. The consultation subgroup has also contributed to the drafting of information leaflets for patients and their relatives in the trial. PPI group members are established within the research governance committees for the trial. PPI group members are also networked to local and national PPI groups and this is an additional resource that we have used to inform our recruitment processes. A dissemination subgroup will coordinate local and national public dissemination activities.

## Ethics and dissemination

The data from the REVAKI-2 study will be available for further ethically approved research studies. The findings will be disseminated by usual academic channels, including presentation at international meetings, as well as by peer-reviewed publications and through patient organisations and newsletters to patients, where available. As the study evaluates technology that is already ubiquitous in high-risk cardiac surgery, we do not predict that there will be commercially exploitable findings from this study.
