# Supplementary material for: Effect of sildenafil (Revatio) on postcardiac surgery acute kidney injury: a randomised, placebo-controlled clinical trial: the REVAKI-2 trial protocol
Source: Open Heart. 2018 Oct 18;5(2):e000838. doi: 10.1136/openhrt-2018-000838 (PMC6196934; doi:10.1136/openhrt-2018-000838)
Supplement: Supplementary data [file openhrt-2018-000838supp001.docx]

**Table 2. Secondary outcomes and definitions**

| Outcome | Definition/method of Verification |
| --- | --- |
| Serum Creatinine | Measured at 6 weeks post-operatively |
| AKI | Defined according to the KDIGO criteria defined as a rise in serum creatinine of >26µmol.l^-1^ within 48 hours or a doubling of the serum creatinine within 7 days of surgery.^1^ |
| Biomarkers of AKI | Measurement of urine NGAL at baseline and at 24 hours post-surgery. We will also calculate absolute change from baseline for serum creatinine. eGFR will be estimated in all patients at 6 weeks post-surgery using the Modification of Diet in Renal Disease equation.^2^ |
| Inflammatory Organ Injury, Sepsis or Death | - Sepsis will be defined as antibiotic treatment for suspected infection, ***and*** *th*e presence of SIRS within 24 hours prior to start of antibiotic treatment where SIRS is defined as ≥ 2 of the following conditions: temperature > 38^o^C or < 36^o^C; heart rate > 90 beats / min; respiratory rate > 20 breaths / min or PaCO2 < 32 mmHg; white blood cell count > 12,000 / mm^3^ or < 4,000 / mm^3^, **or** antibiotic treatment for wound infection. - Acute lung injury, defined as PaO2/FiO2 ratio <300mmHg and CPAP/PEEP of 5 cmH2O (490 kPa).^3^ - Low cardiac output, defined as new intra-or postoperative intra-aortic balloon pump insertion or a cardiac index of <2.2 L · min^−1^ · m^−2^ refractory to appropriate intravascular volume expansion after correction or attempted correction of any dysrhythmias, or the administration of the inotropes enoximone, milrinone or levosimendan. - Stroke; diagnosed by brain imaging (CT or MRI), in association with new onset focal or generalized neurological deficit (defined as deficit in motor, sensory or co-ordination functions) - Acute liver injury will be defined as an acute derangement of liver enzymes three times the upper limit of normal, or an acute derangement of liver enzymes associated with progressive hypoglycaemia and lactate acidosis, or a serum amylase concentration >1000IU/L from daily blood samples. - Acute intestinal injury will be defined a radiological, operative or post-mortem evidence of gut ischaemia. |
| Injury Score | The Multiple Organ Dysfunction Score ^4^ will be calculated at baseline, admission to ICU, 24, 48, 72 and 96 hours post-surgery. |
| Bleeding and Transfusion | - Blood loss at 6 hours postoperatively. - The total number of units of RBC and other blood components transfused during the operative period and post-operative hospital stay will be recorded |
| Drug Reactions | Vital sign measurements during and after drug administration to document;   - Hypotensive reactions and interventions - Allergic Reactions. |
| Other adverse events | Other expected and unexpected adverse events not listed above. |
| Hospital stay and cumulative resource use | Time until extubation, discharge from HDU and discharge from hospital will all be measured from the start of surgery. Deaths will be censored at time of death. |
| Myocardial Injury | Measurement of Serum Troponin I at baseline and at 6-12 and 48 hours post-surgery. |
| Inflammation | Platelet and leucocyte activation will be assessed in blood samples collected at baseline and then at 6 and 48 hours post-surgery using flow cytometry. In addition, pulmonary leucocyte activation will be assessed in leucocytes harvested from pulmonary aspirates at 4-8 hours post-surgery. |
| Endothelial injury | Markers of endothelial activation will be measured in blood samples taken at baseline and then at 6-12hrs and 48 hours post-surgery using flow cytometry. Regional endothelial dysfunction will be measured as the reactive hyperemia peripheral arterial tonometry (RH-PAT) index using the Endo-PAT 2000 (Itamar Medical Ltd., Caesarea, Israel).^5^ Global endothelial dysfunction will also be measured indirectly as the measured time to resolution of oxygen debt defined as the period of time from the end of surgery until the measured serum arterial lactate level falls below 2.5 mmol/L. |

**REFERENCES**

1. Kidney Disease: Improving Global Outcomes. Clinical practice guideline on AKI., 2012

2. Levey AS, Bosch JP, Lewis JB, et al. A more accurate method to estimate glomerular filtration rate from serum creatinine: a new prediction equation. Modification of Diet in Renal Disease Study Group. *Ann Intern Med* 1999;**130**(6):461-70.

3. Force ADT, Ranieri VM, Rubenfeld GD, et al. Acute respiratory distress syndrome: the Berlin Definition. *JAMA* 2012;**307**(23):2526-33. doi:10.1001/jama.2012.5669.

4. Marshall JC, Cook DJ, Christou NV, et al. Multiple organ dysfunction score: a reliable descriptor of a complex clinical outcome. *Crit Care Med* 1995;**23**(10):1638-52.

5. Bonetti PO, Pumper GM, Higano ST, et al. Noninvasive identification of patients with early coronary atherosclerosis by assessment of digital reactive hyperemia. *J Am Coll Cardiol* 2004;**44**(11):2137-41. doi:10.1016/j.jacc.2004.08.062.
